# Application of Asymmetrical Flow Field-Flow Fractionation for Characterizing the Size and Drug Release Kinetics of Theranostic Lipid Nanovesicles

**DOI:** 10.3390/ijms221910456

**Published:** 2021-09-28

**Authors:** Paulina Skupin-Mrugalska, Philipp A. Elvang, Martin Brandl

**Affiliations:** 1 Department of Inorganic & Analytical Chemistry, Poznan University of Medical Sciences, Grunwaldzka 6, 60-780 Poznan, Poland; 2 Drug Transport & Delivery Group, Department of Physics, Chemistry & Pharmacy, University of Southern Denmark, Campusvej 55, DK-5230 Odense M, Denmark; philippelvang3@gmail.com (P.A.E.); mmb@sdu.dk (M.B.)

**Keywords:** asymmetrical flow field-flow fractionation, in vitro release, theranostic, liposomes, microfluidic method

## Abstract

Liposome size and in vitro release of the active substance belong to critical quality attributes of liposomal carriers. Here, we apply asymmetric flow field-flow fractionation (AF4) to characterize theranostic liposomes prepared by thin lipid film hydration/extrusion or microfluidics. The vesicles’ size was derived from multi-angle laser light scattering following fractionation (AF4) and compared to sizes derived from dynamic light scattering measurements. Additionally, we adapted a previously developed AF4 method to study zinc phthalocyanine (ZnPc) release/transfer from theranostic liposomes. To this end, theranostic liposomes were incubated with large acceptor liposomes serving as a sink (mimicking biological sinks) and were subsequently separated by AF4. During incubation, ZnPc was transferred from donor to acceptor fraction until reaching equilibrium. The process followed first-order kinetics with half-lives between 119.5–277.3 min, depending on the formulation. The release mechanism was postulated to represent a combination of Fickian diffusion and liposome relaxation. The rate constant of the transfer was proportional to the liposome size and inversely proportional to the ZnPc/POPC molar ratio. Our results confirm the usefulness of AF4 based method to study in vitro release/transfer of lipophilic payload, which may be useful to estimate the unwanted loss of drug from the liposomal carrier in vivo.

## 1. Introduction

The chief medicine regulatory agencies, the European Medicines Agency (EMA) and the United States Food and Drug Administration (FDA), give general definitions of liposomes, as follows: liposomes are artificially prepared vesicles composed of a bilayer and/or a concentric series of multiple bilayers separated by aqueous compartments formed by amphipathic molecules such as phospholipids that enclose a central aqueous core [1,2]. Active molecules (e.g., therapeutic, imaging, and/or targeting agents) can be incorporated in the lipid bilayers and/or encapsulated in the internal aqueous core; thus, liposomes are suitable carriers for theranostic agents that embrace various precision medicine tools for targeted therapies combining diagnosis, treatment planning, drug delivery, and response assessment [3,4]. Current regulatory guidelines on liposome drug products also define the physicochemical properties that should be characterized and critical quality attributes, such as vesicle size distribution and morphology, that need to be controlled [1,2,5]. Various analytical techniques are commonly used for liposome characterization depending on the analyzed parameter [6,7,8,9].

In recent years, asymmetric flow field-flow fractionation (AF4) has been gaining attention as a technique, which allows thorough and detailed analysis of the size and size distribution of nanoparticle dispersions over a wide range of particle sizes irrespective of the polydispersity of the sample. It thus is suited for quality control of nanomaterials, including (phospho)lipid-based nanocarriers and innovative systems such as lipid nanoparticles [10,11,12].

Field flow fractionation is a family of separation techniques useful for the separation of sub-micron particles over a wide range of sizes while providing high resolution. It is characterized by gentle separation conditions and a broad working range. With this method, unlike conventional chromatography, there is no stationary phase. The particles (or macromolecular solutes) are separated by applying a force field perpendicular to the forward flow of the particles in a ribbon-like channel. In flow field-flow fractionation, an adjustable crossflow is applied perpendicularly to the main channel flow, leading to particle separation dependent on their hydrodynamic sizes [13,14]. The Brownian motion of smaller particles is to a smaller extent compensated for by the applied crossflow and thus equilibrate/travel further away from the accumulation wall. Due to the longitudinal parabolic flow profile in the channel, smaller particles will elute faster than larger ones closer to the semipermeable membrane (accumulation wall) [13]. The fractionation system can be coupled to different detectors, mostly comprising multi-angle laser light scattering (MALLS), differential refractive index (dRI), or UV/VIS. Such setups have been useful in determining size distribution and physical properties of nanocarriers, drug-carrier interactions (drug loading, drug release), carrier-biomolecule interactions, and evaluating the structural organization of nanocarriers [11,14,15,16].

In vitro drug release study is an important step in the characterization of the functionality of drug formulations [17,18]. Drug release from nano-sized carriers can be assessed by three categories of methods, including “sample and separate”, continuous flow, and dialysis membrane methods [17]. However, all of the aforementioned techniques demonstrate poor in vivo correlation in the case of lipophilic molecules as the applied conditions do not reflect physiological conditions. The main factor affecting the kinetics of lipophilic drug release in vitro is the solubility of the drug in the release medium. The obstacle mentioned above can be overcome by supplementing the release medium with additives capable of dissolving the hydrophobic substance such as surfactants [19], serum albumin [20], or organic solvents [19,21]. These were categorized as “external sink methods” [17,18]. Recently, different lipophilic acceptors have been added to the release media for in vitro release assessment of lipophilic substances from nanoparticles. For example, Petersen et al. [15] used lipophilic acceptor emulsion droplets to investigate the transfer of fluorescent dye by a flow cytometric method. Another approach relied on a transfer setup in which a lipid nanoparticle suspension incorporated into small calcium-alginate hydrogel microbeads served as the lipophilic acceptor for drugs released from lipid nanoemulsions [22,23]. Further, large acceptor liposomes were employed by Hinna et al. [10,14,16,24] to study the transfer and release of hydrophobic porphyrin (meso-tetra(hydroxyphenyl)porphyrin) and other dyes from liposomes, employing AF4 as a separation technique.

We developed theranostic liposomes to transport a contrast agent (a hybrid of a phospholipid and gadopentetic acid) for magnetic resonance imaging (MRI) and a therapeutic photosensitizing agent (PS) for photodynamic therapy (PDT) of cancer [25,26]. Zinc(II) phthalocyanine (ZnPc) serves as a model PS in our delivery system. It is a second-generation PS developed for PDT of various tumors and benign conditions because of its advantageous chemical and photophysical properties [27,28,29].

The method of PDT involves the use of a PS, usually a macrocyclic dye that is relatively selectively accumulated in abnormal or neoplastic cells. Most PSs are insoluble in water; therefore, they require a delivery vehicle to enhance solubility and bioavailability. Liposomes have been most commonly used as carriers for PSs, and that strategy has been successfully translated into the clinic [30]. The PS accumulated in the tumor is activated with a specific wavelength of light, matching the unique absorption characteristics of that particular PS, usually using a laser. This results in tumor necrosis via several mechanisms, including the production of reactive oxygen species and vascular shutdown to the tumor [31]. The clinical application of PDT is usually limited by the ability of light to penetrate the human body, so it is used to treat skin malignancies, esophagus, head and neck cancer, etc. [32]. The presented theranostic liposomal system was designed and initially studied in vitro in a head and neck cancer model [26], assuming intravenous administration and application of PDT as a monotherapy or adjuvant intraoperative PDT following tumor resection. In the paper [26], we showed that phototoxicity of ZnPc-loaded theranostic liposomes exceeds free ZnPc in the head and neck cancer model. The advantage of PDT over other conventional modalities such as surgery, radiation, and chemotherapy is that it is a minimally invasive treatment technique that lacks systemic toxicity yet results in selective tumor destruction with normal tissue preservation. This advantage is of particular importance for cancers of the head and neck, where excessive tissue loss results in significant functional impairment. In addition, PDT can be easily combined with chemotherapy, ionizing radiation, or surgery [32].

Drug delivery nanomaterials, mostly liposomes, have been shown to be suitable carriers for head and neck cancer treatment [33]. That strategy was also investigated in several clinical trials [34,35]. However, the achievement of the optimal combination of physicochemical parameters to specifically target the tumor site and control drug release has been identified as key factors that hamper the translation of nanoformulations into therapy [33]. To address the mentioned challenges in head and neck cancer therapy and use the benefits of PDT, we aimed to develop a liposome-based system that ensures the efficient delivery of ZnPc to the tumor site and offers the opportunity for subsequent monitoring by MRI. To ensure prolonged circulation in the bloodstream and decrease the risk of premature clearance by the liver, one of the formulations was decorated with polyethylene glycol chains. We expect that the nature of the liposomal vesicles and their size characteristic will prevent the unwanted loss of ZnPc from the theranostic liposomal carrier in vivo and result in effective delivery system.

In our recent paper [26], we have described theranostic nanoliposomes characterized by dynamic light scattering (DLS) measurements and transmission electron microscopy (TEM). The evident limitations of these “bulk” measurement techniques encourage the addition of a fractionation step to analyze potential multiple particle fractions.

In this study, AF4-MALLS was utilized to analyze the size distribution of recently developed “limit-size” theranostic liposomes prepared by the microfluidic method [26]. This was to compare with the previously obtained results from DLS and to analyze the in vitro release of the active photosensitizing agent—zinc phthalocyanine (ZnPc)—from the nanovesicles.

It was demonstrated by Hinna et al. [14,16,24] that the kinetics of model drug transfer from small-donor liposomes (DL) to large acceptor liposomes (AL), which mimics the drug transfer to various biological sinks (mostly plasma proteins and lipoproteins), can be determined by both online and offline analysis. That means that drug concentration can be analyzed directly during AF4 separation using a suitable detector (e.g., UV/vis detector for sensitive chromophores), which refers to online analysis. Otherwise, the separated fractions have to be eluted from the channel, collected, and then analyzed offline by an appropriate analytical method, such as chromatography (HPLC) or HPLC method coupled to mass spectrometry.

Therefore, the present study aims to adapt the previously established AF4-MALLS-based assay to study the size of theranostic nanovesicles and determine the transfer of a hydrophobic photosensitizing agent, ZnPc and thus estimate its release kinetics from theranostic liposomes. We followed incubation conditions, briefly DL to AL mass lipid ratio, injection volume into AF4 channel, applied by Hinna et al. [16]. However, flow and crossflow parameters were optimized to provide the desired separation of DL and AL. Additionally, we established an offline method for the ZnPc concentration analysis. In contrast to Hinna et al. [16], we could not perform online ZnPc analysis due to the limited spectral range of the UV/vis detector employed.

## 2. Results

### 2.1. Liposome Size Characterization

We have recently described the characteristics of theranostic liposomes prepared by thin lipid film hydration followed by extrusion (TLH+extrusion), (F4M1), and microfluidic method (F4M2, F4M3, F4M4) [26]. Here, we additionally present the new pegylated analog formulation of F4M2, named F5M2, and acceptor liposomes (AL) needed for the transfer study (Table 1). The pegylated liposomes were obtained with a perspective of in vivo application of the theranostic liposomes in head and neck solid tumors. The incorporation of amphipathic poly(ethylene glycol) (PEG) conjugates in liposome bilayers has been shown to prolong liposome circulation. Such long-circulating liposomes are able to accumulate in solid tumors [36].

The ZnPc load was quantified with UV/VIS measurements and found to be in the range of 8.03–22.02 µg/mL in the liposomal solution, corresponding to 0.14–0.38 mol%. The lipid content in the theranostic liposomes used in the study was 2.8–5.8 mg/mL, and the samples consisted of unilamellar, homogeneous vesicles [26]. The AL contains large vesicles possessing two or more concentric bilayers or of multivesicular structure [10]. The content of lipid in the AL dispersion was 9.8 mg/mL (Table 1).

### 2.2. Characterization of Liposome Size by DLS and AF4-MALLS

**Figure 1:**
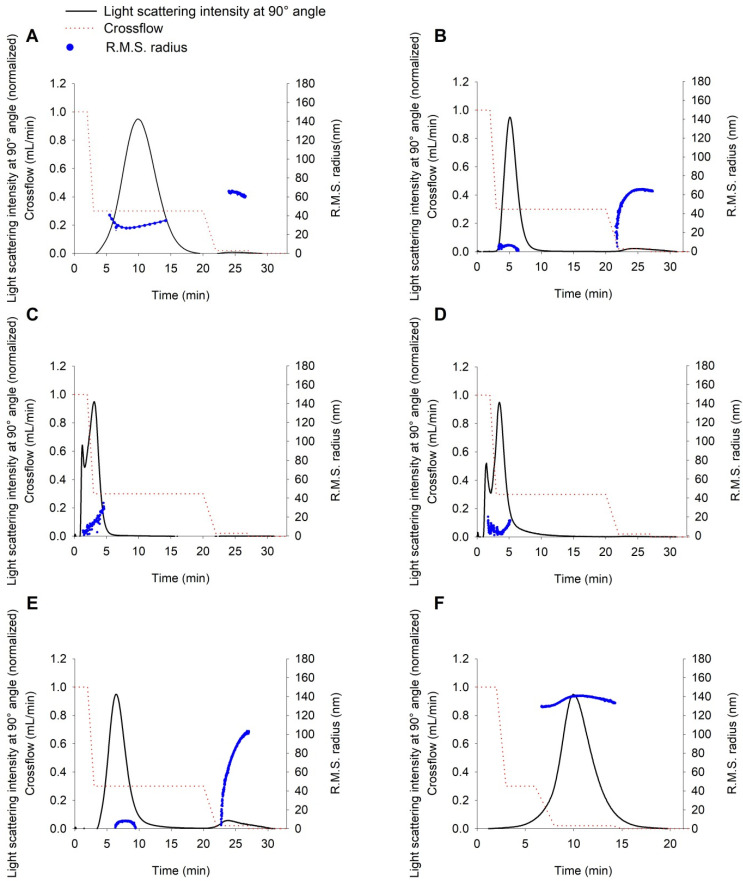
Theranostic liposome sizing by AF4 with online MALLS. Fractograms of light scattering intensity at 90° angle (black) with associated R.M.S. radius profiles (blue) and applied crossflow (red dotted line) obtained for theranostic liposomes: (**A**)—F4M1 (TLH+extrusion), (**B**)—F4M2 (FRR 3:1, TFR 12 mL min^−1^), (**C**)—F4M3 (FRR 5:1, TFR 12 mL min^−1^), (**D**)—F4M4 (FRR 3:1, TFR 8 mL min^−1^) and (**E**)—pegylated F5M2 (FRR 3:1, TFR 12 mL min^−1^), (**F**)—acceptor liposomes.

All the liposomal samples were measured by DLS and were fractionated using the frit-inlet channel of the AF4 system to evaluate the size of both DL and AL. The fractionation step with AF4 revealed one fraction in DL, samples F4M3, F4M4, and AL (Figure 1C,D,F). On the contrary, in the fractograms of samples F4M1, F4M2, and F5M2, the one prevailing fraction was eluted, followed by a profoundly smaller fraction containing larger particles. To confirm that any occurring peak is not related to the presence of any contamination, a blank sample was injected before each run (data not shown).

**Figure 2:**
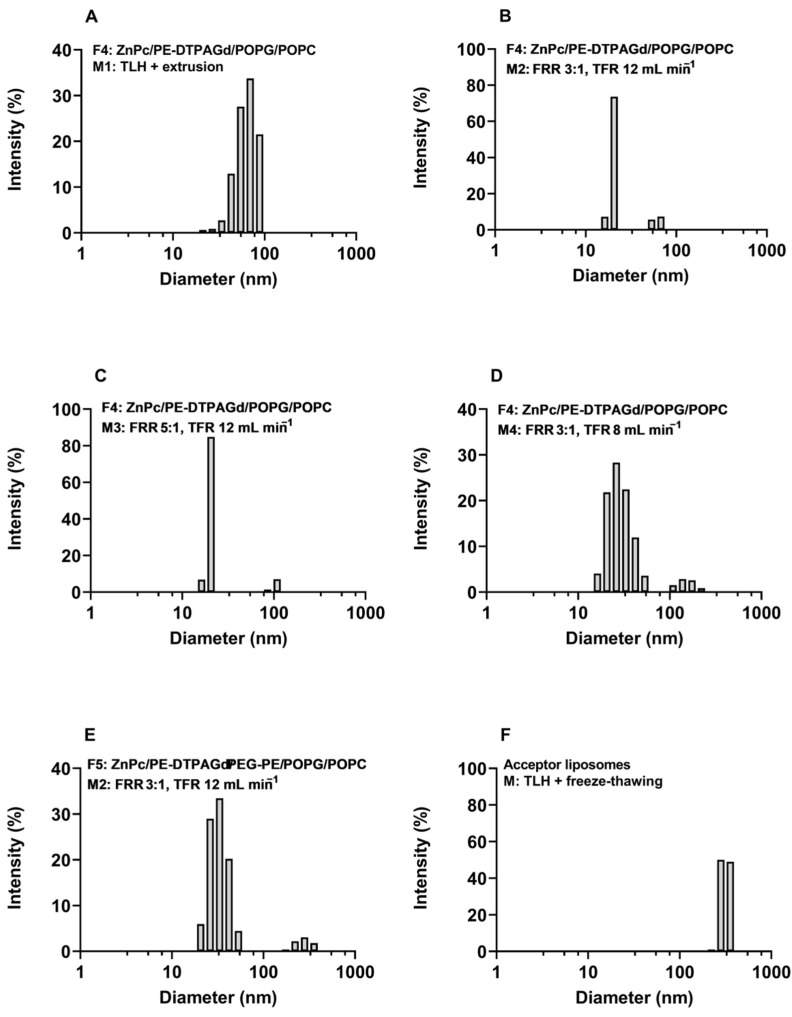
Regularization analysis graphs obtained from DLS measurements of donor liposomes: (**A**)—F4M1, (**B**)—F4M2, (**C**)—F4M3, (**D**)—F4M4, (**E**)—F5M2, and acceptor liposomes—(**F**).

DLS measurements showed the presence of one population in samples F4M1 and AL (Figure 2A,F). As indicated in AF4-MALLS results obtained for sample F4M1 (Figure 1A and Table 2), the two vesicle populations’ sizes differ by a factor of 2 (diameter 60 nm vs. 120 nm). The DLS technique cannot resolve this as separate populations and, as expected, shows only one peak (Figure 2A). In all other samples (F4M2, F5M2, F4M3, F4M4), two peaks occurred in the regularization analysis graphs presented in Figure 2B–E. Among charts in which two peaks are present (Figure 2), the percentage of intensity for the second peak is below 10% (F5M2, F4M3, F4M4) and only exceeds this value in the sample F4M2 (Table 2). Notably, for each sample, the diameter of the dominant peak (meaning the highest intensity) read from the regularization analysis graph has the closest values to the hydrodynamic diameters (z_av_) resulting from the cumulant analysis. The hydrodynamic diameter determined by DLS correlates well with the geometric diameter obtained from AF4-MALLS, as presented in Table 2.

AL’s morphology is different from DL, as we reported in the previous study [10], and the sample contains almost exclusively oligo-, multilamellar, and multivesicular liposomes. For that reason, the application of the hollow-sphere model in AF4-MALLS analysis gives underestimated results [10]. The results obtained using the solid-sphere model results for AL size analysis have the best fit to the raw scattering data and provide more reliable information.

The results of size and size distribution measurements of theranostic and acceptor liposomes by AF4-MALLS and DLS are presented in Table 2 and Supplementary Material Table S1.

Peak 1 of the donor theranostic liposomes prepared by TLH+extrusion exhibited a mean particles size of 67.1 nm (D_z_) with a size distribution of 56.8–72.5 nm (D_10_–D_90_). The geometric diameters of DL obtained by microfluidics are 2–3 times smaller than diameters of vesicles prepared by TLH+extrusion. The mean particles size of peak 1 in the DL resulting from the microfluidic method was in the range of 23.7–31.0 nm (D_z_) and the size distribution 18.0–30.3 nm (D_1**0**_) and 30.8–58.9 nm (D_90_). Second peaks (Peak 2) observed in the fractograms of samples F4M1, F4M2, F5M2 are characterized by the mean particle size of 123.2–196.9 nm (D_z_) and the distribution ranging from 98.5–153.9 nm (D_10_) to 148.3–212.5 nm (D_90_). All the theranostic liposomes have narrow size distribution and exhibit PDI values acceptable for the homogeneous colloidal system, ranging from 0.084 to 0.235.

The preparation method of AL adapted from Hinna et al. [10] leads to vesicles with diameters of approx. 300 nm. Indeed, the applied TLH+freeze–thawing resulted in AL with z_av_ of 302.9 nm and narrow size distribution (PDI = 0.111). The size measurements performed by AF4-MALLS revealed that the fraction was characterized by a mean particle size of 276.2 nm (D_z_) and size distribution between 237.2 nm (D_10_) and 276.8 nm (D_90_).

Theranostic liposomes, which will serve as the DL in the further transfer study, exhibit a mean particle size sufficiently different from the mean particle size of the AL. Both DL and AL have narrow size distributions as required to avoid overlap of the donor and acceptor fractions.

### 2.3. Transfer Study

**Figure 3:**
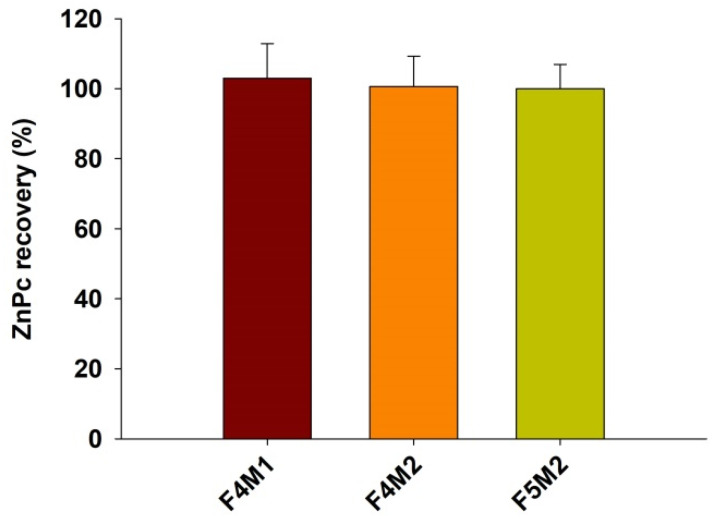
ZnPc recovery following fractionation of donor theranostic liposomes F4M1, F4M2, and F5M2, fraction collection from the AF4 channel, and up-concentration in relation to the initial content in the injected volume of donor vesicles.

For the transfer study, three liposome samples were used: F4M1, F4M2, and pegylated F5M2. All three samples revealed the presence of two fractions in the AF4-MALLS fractograms (Figure 1). Even though the second fraction seems to be negligible, before the transfer study, we analyzed the possible interference of the second fraction (Peak 2) present in the fractograms of the samples on the ZnPc concentration determination in the acceptor fraction. For that purpose, we fractionated donor liposomes F4M1, F4M2, and pegylated F5M2 using the AF4-MALLS separation method 3. Then, we collected the fractions of DL corresponding to Peak 1 and Peak 2 separately and the total DL (fractions corresponding to Peak 1 and Peak 2) and subsequently determined the ZnPc content in each fraction. The ZnPc content in the samples corresponding to the fraction of Peak 2 did not give any absorption characteristic to ZnPc dye (or the content was below the quantification limit). Significantly, there were no differences between the ZnPc content in the total collected fraction of DL and samples collected in the time range corresponding to Peak 1. The calculated contents (F4M1—103.0 ± 9.9, F4M2—100.6 ± 8.7, F5M2—100.0 ± 6.9) showed the approx. 100% recovery of ZnPc following the AF4-MALLS fractionation in relation to the ZnPc content in the injection volume (Figure 3).

**Figure 4:**
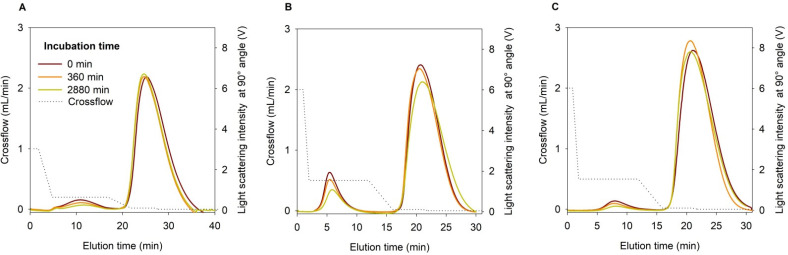
Selected fractograms at the incubation time points 0 min (maroon), 360 min (orange), and 2880 min (lime green) plotted during AF4-MALLS separation of donor liposomes (**A**—F4M1, **B**—F4M2, **C**—F5M2) and acceptor liposomes, following incubation at 37 °C and with crossflow gradient of AF4 separation Method 3 (**A**) and Method 4 (**B**,**C**).

The above results confirm that the impact of peak 2 on the ZnPc content in the AL during the ZnPc transfer study is not significant, and the selected separation conditions are suitable for the ZnPc transfer study. The latter can be further confirmed by the fractograms presented in Figure 4.

Theranostic DL and AL vesicles were mixed at 1:0.8 lipid mass ratios for transfer study. The prepared mixtures were incubated and subsequently fractionated by AF4 after incubation periods ranging from 60 min to 48 h. We established two AF4 separation methods of F4M1 (Method 3) and F4M2, F5M2 (Method 4) donor liposomes from AL. Different crossflow gradient conditions (Table 3) were required to separate F4M1 characterized by z_av_ of 58.8 nm from AL than much smaller F4M2 (z_av_ = 24.2 nm) and pegylated F5M2 (z_av_ = 32.8 nm). Figure 4 presents fractograms of three incubation mixtures, varying in the type of DL (F4M1, F4M2, and F5M2), for selected incubation time points (0 min, 6 h, and 48 h). In all cases, both fractions appeared well separated (Figure 4) with a gap of a few minutes between peaks, thus allowing the collection of both fractions. Baselines were registered for blank injection of TRIS buffer under flow conditions, i.e., crossflow used to separate donor and acceptor fractions (Supplementary Material Figure S1).

**Figure 5:**
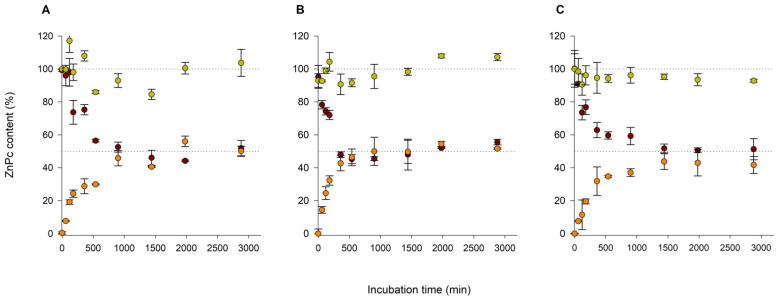
ZnPc transfer from donor liposomes (**A**)—F4M1, (**B**)—F4M2, (**C**)—F5M2 to acceptor liposomes during incubation for 48 h at 37 °C. Graphs present the amount of ZnPc relative to the initial content in incubation mixtures determined in the donor fraction (maroon), acceptor fraction (orange), and the total determined content (lime green) plotted over the incubation time. Donor and acceptor liposomes were separated by AF4-MALLS, fractions were collected and up-concentrated, and ZnPc content was determined spectrophotometrically.

Our focus was to perform offline analysis of ZnPc by UV/vis measurements in collected DL and AL fractions. The UV/vis analysis required up-concentration of collected fractions due to pronounced dilution of injected volume during the AF4-based transfer experiment. ZnPc was transferred from donor to acceptor vesicles reaching the transfer equilibrium at 48.7% (F4M1), 46.7% (F4M2), and 53.1% (F5M2) after ca. 17, 48, and 23 h, respectively (Figure 5, Table 4). The constant rate differed between formulations and increased in the order F4M2 < F5M2 < F4M1 (Table 4).

### 2.4. Mathematical Modeling of ZnPc Release from DL

**Figure 6:**
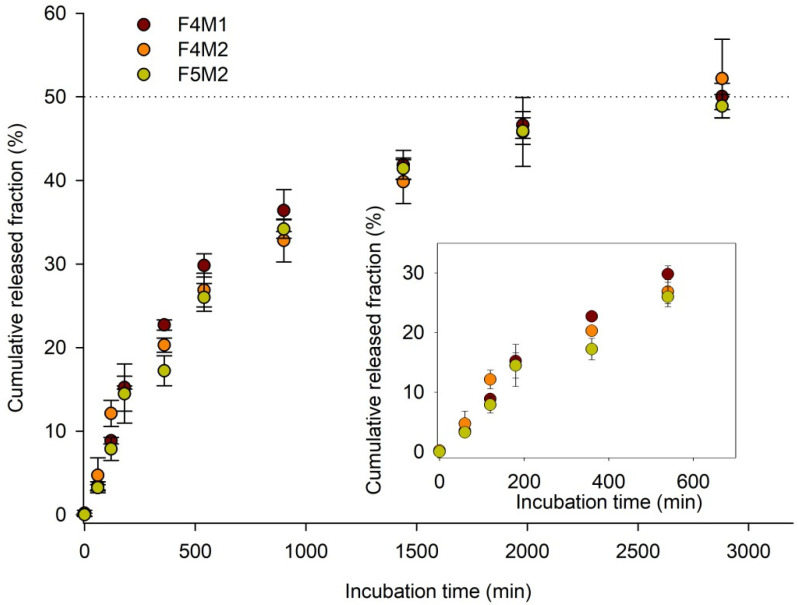
Cumulative released fraction of ZnPc from theranostic donor liposomes: F4M1 (maroon), F4M2 (orange), and F5M2 (lime green) during incubation at 37 °C for 48 h. Inserted graph shows the initial ZnPc transfer and release during the first 10 h of the experiment.

To better understand the mechanism underlying ZnPc release from theranostic DL, the cumulative released fraction from DL was calculated for each studied DL formulation (Figure 6). Multiple comparison analysis showed that there are no statistically significant differences between analyzed release profiles presented in Figure 6.

Subsequently, data were fitted to the Higuchi, Korsmeyer-Peppas, and Peppas-Sahlin mathematical models using DDSolver software [37].

The mechanism by which the drug release is governed can be determined by statistical analysis of the first 60% of all release curves and is based on the higher r^2^ and lower Akaike information criterion (AIC) [38]. As shown in Table 5, r^2^ is the highest while AIC is the lowest for the Peppas–Sahlin model. The Peppas–Sahlin model is a release kinetics model that assumes two contribution mechanisms, diffusional and relaxational, in a drug release process. Therefore, the results indicate that the release of the ZnPc from all studied DL is governed by the combination of Fickian diffusion and liposome relaxation.

## 3. Discussion

Particle size affects different factors such as drug loading and release behavior, cellular uptake, intracellular fate, and pharmacokinetic properties [36,39]. The size of studied theranostic donor liposomes is within the typical range (10–200 nm) for nanomaterials used for drug delivery. Notably, the vesicles prepared by microfluidics were smaller, with z_av_ ranging from 22.1 nm to 32.8 nm, than those prepared by TLH+extrusion with a z_av_ of 58.8 nm.

The range of 10–200 nm is mostly defined by the mononuclear phagocyte system (MPS) uptake cut-off and the opportunity to take advantage of enhanced permeation and retention effect in nanoparticle delivery to the solid tumors [40,41]. For a nanoparticle to exhibit prolonged circulation and the optimized EPR effect, the lower particle size limit is 5–10 nm, which is the renal filtration cut-off size [39,42]. A second lower limit is imposed by liver filtration, as vascular fenestrations in the liver are 50–100 nm, and particles smaller than 50 nm will interact with hepatocytes [39]. Litzinger et al. [36] showed that small pegylated liposomes (d < 70 nm) were more rapidly cleared by the liver from the circulation than the larger long-circulating pegylated liposomes (d~150–200 nm). The aforementioned suggested that the more rapid clearance by the liver would expectedly reduce the opportunity for the smaller liposomes to accumulate in the tumor.

The upper limit of particle size is influenced by two factors: tumor permeability with vascular fenestration varying from 300–600 nm to microns among different tumors [43,44], and splenic filtration trapping particles not exceeding 300–400 nm [39].

These literature data imply that tumor accumulation of theranostic liposomes with diameters profoundly below 70 nm may be susceptible to rapid liver clearance under in vivo conditions. However, Harrington et al. [45] demonstrated the prominent liposome uptake in head and neck cancers, suggesting that those tumors are suitable targets for liposome-based therapies. The above finding seems promising, considering that the theranostic liposomes presented herein are designed to target head and neck carcinomas. Additionally, it has been recently shown that smaller (50 nm) delivery agents may substantially express the improved extravasation, penetration, and retention within the tumor tissue [46,47]. Lee et al. [48] showed that the accumulation of the 25 and 60 nm particles in the liver and spleen were not significantly different. Still, tumor uptake of the 25 nm particles was 2-fold higher relative to the 60 nm particles. Considering the above findings, pegylated theranostic liposomes F5M2 with z_av_ = 32.8 nm seem to have the most suitable properties to provide prolonged circulation, exhibit EPR effect, and present satisfactory accumulation in a targeted tumor; briefly, they may result in good performance under in vivo conditions in the head and neck cancer model.

DLS is the most common method used for the size measurement of liposomes and other colloidal materials. The Brownian motion of particles or macromolecules in suspension causes the laser light to scatter at a frequency dependent on their particle size. Including a time (t) dimension, analysis of these intensity fluctuations yields the velocity of the Brownian motion, and the particle size can be derived using the Stokes–Einstein relationship. DLS allows fast and easy-to-perform analyses; however, larger particles strongly influence the measurement results and complicate the analysis of heterogeneous (size) samples. As a rule of thumb, two populations of particles must differ in average sizes by a factor of 5 for DLS to resolve them as separate populations.

In contrast, AF4 is more time-consuming but offers effective sample fractionation when appropriate flow/separation conditions are established. MALLS raw scattering data with associated software’s analysis provide the molar mass and the radius of gyration (root-mean-square radius) of the solutes [11]. The radius of gyration is related to the geometric radius and the distribution of mass in the particles.

DLS and AF4-MALLS distinguished more than one population of particles in the studied theranostic DL samples, even though the results were mutually exclusive in two examples of samples, F4M3 and F4M4.

Importantly, MALLS as the size detector requires fitting the scattering data obtained by the detector with a suitable model. In our study, we used methods provided by the software based on the theory of light scattering and the assumption on the geometry of the particles, briefly, hollow- and solid-sphere models. The choice of the model was based on the previous analysis of the studied theranostic DL and AL by TEM. Additionally, while we found the hollow-sphere model suitable for size determination of DL, which are small unilamellar vesicles [26], the results were underestimated in the case of large AL, and the solid-sphere model provided a better fit to the scattering data where the particle sizes were comparable with TEM and DLS data. This agrees with previous findings (Hinna et al. [10]) for large acceptor vesicles.

In our experimental setup, the AL mimics biological sinks such as red blood cell membranes and lipoproteins, representing a wide range of particle sizes (from tens to thousands of nm). The AL sample contains almost exclusively oligo-, multilamellar, and multivesicular liposomes [10] with diameters of ca. 300 nm. Hinna et al. [24] showed that equilibrium distribution and transfer rate of membrane-associated compounds from liposomal drug carriers to a model sink (large oligo-,multilamellar/multivesicular liposomes) correlated well with the lipid mass ratio (donor/acceptor) and were independent of vesicle size and lamellarity, indicating that upon transfer, the compounds were readily redistributed from donor to acceptor vesicles, and it is the vesicle-to-vesicle transfer that is rate-limiting.

The compound incorporated within liposomes in our study—ZnPc—is a highly water-insoluble, hydrophobic macrocycle. ZnPc, as a molecule of a highly lipophilic nature, requires a delivery system that constitutes a matrix in which molecules can be solubilized and, as such, delivered for in vitro or in vivo applications. Therefore, ZnPc has been mostly formulated in liposomal form [49,50,51]. Additionally, in our study, ZnPc is embedded within the lipid bilayer of theranostic liposomes. In light of the aforementioned and the compounds’ poor aqueous solubility, we carefully considered the possible ZnPc release setup. De Paula et al. [21] used the “sample and separate” method with *n*-octanol providing external sink conditions. De Souza et al. [19] studied ZnPc release from nanocapsules made of chitosan and/or lipids by dialysis. At the same time, the mixture of buffer (sodium acetate–acetic acid pH 5.4 with 1% SDS) and DMF (30:70 *v*/*v*) served as an external acceptor medium because of its best performance in terms of saturation concentrations of ZnPc (50%) [19].

However, as shown by Reddi et al. [49], ZnPc incorporated within small unilamellar liposomes, once injected into the bloodstream, is almost quantitatively transferred into lipoproteins. Additionally, liposome carriers of ZnPc have been reported to fuse with the lipid matrix of lipoproteins and thus transfer the entrapped drug to lipoproteins [49]. Subsequently, the circulation half-life and biodistribution of the drug are no longer controlled by the liposome carrier. It is thus desirable to design liposomal carriers for ZnPc, which retain the drug or—in other words—reduce the premature loss of drug in the bloodstream.

To this end, we found the procedure established by Hinna et al. [16] to be the best choice for in vitro release/transfer assessment of ZnPc from theranostic liposomes because it may reflect, to some extent, the in vivo process. Our results indicate that the method could be easily adapted to a different liposomal system and active molecule under the pre-requisite that a sufficient size difference between donor and acceptor fraction is ensured. A good fractionation profile was obtained with preserved DL to AL mass lipid ratio (1:0.8) [16] due to optimized selection of AF4 channel, injection volume, and crossflow gradient (Figure 4). The up-concentration of collected fractions and offline determination of the studied compound in DL and AL was more demanding. In contrast to Hinna et al. [16], we could not perform online ZnPc analysis due to the limited spectral range of the UV/vis detector employed.

The transfer kinetics of ZnPc between DL and AL can be described well with a simple exponential function. The determined rate constants of transfer differed between formulations of theranostic liposomes. The obtained data may thus be useful for comparing the performance of different theranostic liposome formulations, more specifically F4M1, F4M2, and F5M2, in terms of release rate.

**Figure 7:**
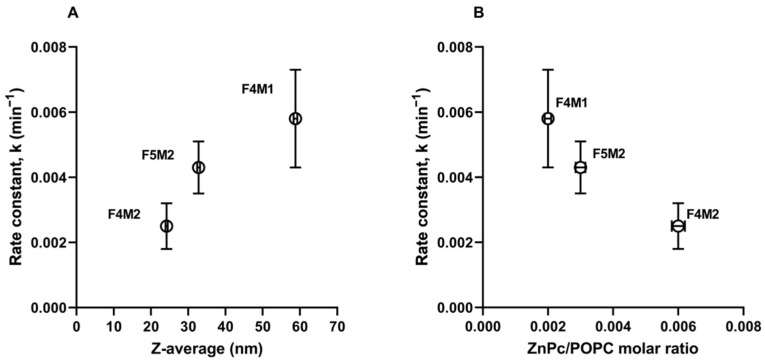
The influence of liposome size (z_av_) (**A**), and ZnPc/POPC molar ratio (**B**) on ZnPc release rate constant k.

The size of the particles is a well-known factor that can affect the release of drug molecules from the carrier. Smaller nanoparticles have a larger surface-area-to-volume ratio and thus can encapsulate less drug and release it faster with the drug being closer to the surface [52,53]. For example, the release rate constant of a lipophilic drug (DB-67—camptothecin analog) for 146 nm liposomes was lower than 103 nm liposomes, meaning DB-67 was retained longer in larger liposomes [54]. However, smaller theranostic liposomes obtained by microfluidics (F4M2, z_av_ = 24.2 nm and F5M2, z_av_ = 32.8 nm) were characterized by higher ZnPc loading (Table 1) and exhibited lower constant rates than larger DL (F4M1, z_av_ = 58.8 nm) prepared by TLH+extrusion. As shown in Figure 7A, the rate constant of ZnPc transfer increased with the size of studied theranostic liposomes. In contrast, the rate constant decreased with the increasing ZnPc-to-POPC molar ratio in the formulation (Figure 7B).

There is a well-founded body of literature regarding design and release characteristics of liposomal drug carriers with water-soluble drugs entrapped within the aqueous core; e.g., Johnston et al. [55,56] have shown that the release properties of vincristine and doxorubicin encapsulated in large unilamellar vesicles (LUV) can be regulated by varying the drug-to-lipid (D/L) ratio and that the half-times for drug release from LUV in vitro can be increased by increasing the D/L ratio. Such behavior is common for drugs that precipitate following liposome accumulation and encapsulation in the internal aqueous core. Therefore, the denser the precipitate and the slower the dissolution rate of the precipitate, the slower the drug release from the liposomes [57].

In contrast, the mechanistic understanding of the release/transfer of drugs embedded in the lipid bilayer of theranostic liposomes, such as ZnPc, is still somewhat limited. Thus, for years, the formulation design of such liposomal drug carriers followed the trial-and-error approach with numerous biodistribution experiments in animals. Only in recent years has it been widely accepted that the key challenge for achieving sufficient accumulation in the target tissue with this type of liposomal drug delivery system is not the biodistribution/targeting of the carrier but the premature loss of drug during transport via the bloodstream.

However, notably in smaller theranostic liposomes (F4M2 and F5M2) with more ZnPc molecules loaded, ZnPc may show a higher tendency to form aggregates that are retained longer in the lipid membrane and are released more slowly. Reshetov et al. [58] observed the enhanced formation of aggregates by 5,10,15,20-tetrakis(3-hydroxyphenyl)-chlorin embedded in liposomes with an increasing drug to lipid ratios. Isele et al. [51] described liposomes containing different content of ZnPc monomeric fraction, which affected pharmacokinetic properties of ZnPc in mice. The aggregation of ZnPc in the liposomes (i) increased the clearance rate of the dye from plasma, (ii) lowered the maximal dye concentration in tumor tissue, and (iii) increased the maximal dye concentration in the liver [51]. The direct comparison of the in vitro release data to in vivo data is also not always possible. Noteworthy, the half-times of ZnPc loss from donor theranostic liposomes, t_1/2_ = 119.5–277.3 min (depending on the DL formulation), corresponded to the estimates of the half-lives of ZnPc distribution in mice t_1/2α_ = 126–288 min, which were dependent on the content of ZnPc monomeric fraction in the studied liposomes [51].

Another possible explanation for prolonged retention of ZnPc in smaller theranostic liposomes is that a hydrophobic component placed in the vicinity of phospholipid chains may stiffen the unsaturated phospholipid chains, similarly to cholesterol [59,60]. Unsaturated phospholipid, POPC, is the main component of theranostic liposomes. Therefore, we hypothesize that increased content of ZnPc incorporated into theranostic liposomes F4M2 and F5M2 may cause stiffening of the structure in some locations of the liposomal membrane, resulting in prolonged retention in the vehicles and slower transfer to acceptor liposomes in comparison to theranostic liposomes prepared by extrusion (F4M1) with profoundly lower loading of ZnPc.

Various mathematical models for drug release have been proposed and explored for liposomes depending upon the nature of the drug and composition of lipids for better correlation in in vitro–in vivo outcomes to assure improved safety and efficacy [61]. A mathematical model fitting to in vitro data demonstrated that two mechanisms, Fickian diffusion and liposome relaxation, contributed to the ZnPc release from theranostic liposomes. Feuser et al. [62] recently showed that ZnPc was released from liposomes composed of soybean phosphatidylcholine with a dominant fraction of saturated phospholipids. Feuser et al. [62] performed a release study by the “sample” method (no separation step was mentioned) with 0.5% sodium dodecyl sulfate in the release medium to provide sink conditions. The process was controlled by a typical diffusion mechanism based on Korsmeyer–Peppas model fitting. However, the results cannot be directly compared to ours because of different liposome compositions, experimental setups, and various models chosen for mathematical modeling.

In summary, the application of the AF4-based method for in vitro release and transfer study of ZnPc from theranostic liposomes offered the opportunity to combine the advantages of the “sample and separate” method (fractionation in AF4 system) with the external sink method due to the usage of large acceptor liposomes. Thus, it was possible to compare the release rate of the active compound from different liposomal formulations.

## 4. Materials and Methods

### 4.1. Materials

1-palmitoyl-2-oleoyl-*sn*-glycero-3-phosphocholine (POPC), 1-palmitoyl-2-oleoyl-*sn* -glycero-3-phospho-(1′-rac-glycerol) sodium salt (POPG), ammonium salt of 1,2-dipalmitoyl-*sn*-glycero-3-phosphoethanolamine-N-[methoxy(polyethylene glycol)-2000] (PEG2000-PE), and 1,2-dipalmitoyl-*sn*-glycero-3-phosphoethanolamine N-diethylenetriaminepentaacetic acid gadolinium (III) salt (PE-DTPAGd), were purchased from Avanti Polar Lipids Inc. (Alabaster, AL, USA). Zinc phthalocyanine (ZnPc) was purchased from Sigma Aldrich (St. Louis, MI, USA).

### 4.2. Thin Lipid Film Hydration Followed by Extrusion (TLH+Extrusion)

Theranostic liposomes (F4M1) were prepared by conventional thin-film hydration method and subsequently extruded 21 times through polycarbonate membranes (Whatman, Kent, UK) with a pore diameter of 100, 50, and 30 nm, using a syringe extruder (LiposoFast Basic mini extruder, Avestin Inc., Ottawa, ON, Canada). A detailed description of the procedure can be found here [26]. Unbound material was separated from liposomes by fast ultrafiltration using Amicon Ultra 2 mL centrifugal filters with 50 kDa MWCO (Merck KGa, Darmstadt, Germany). Liposome samples were stored at 2–8 °C, protected from light.

### 4.3. Micromixer and Microfluidic Preparation of Liposomes

Samples F4M2, F5M2, F4M3, F4M4 were prepared by microfluidics. POPC and POPG were dissolved in ethanol to obtain 25 mg/mL solution. ZnPc was dissolved in DMF, giving 0.4 mg/mL stock solution. PE-DTPAGd (10 mg/mL) was dispersed in ethanol. Liposomes were prepared using a microfluidic micro-mixer NanoAssemblrTM (Benchtop, Precision NanoSystems Inc., Vancouver, BC, Canada) equipped with a microfluidic cartridge (dimension 6.6 cm × 5.5 cm, and 0.8 cm height). The appropriate volume of ZnPc solution was added to POPC/PG, giving an organic solution containing less than 20% of DMF. At the same time, ethanolic dispersion of PE-DTPAGd was diluted in 10 mM TRIS buffer. Both fluids were pumped into the two inlets of the microfluidic micro-mixer using disposable syringes at a total flow ratio (TFR) of 8 and 12 mL/min and at different aqueous/organic flow rates ratios (FRR) 1:3 or 1:5. The organic solvent was removed by centrifugal filtration using Amicon Ultra centrifugal filters with 50 kDa MWCO. Briefly, liposomes were added to the Amicon filters and centrifuged at ca. 6000 rcf for 45 min. Afterward, 10 mM TRIS buffer was added to the filtered formulation and spun again at 6000 rcf for 45 min. The procedure was repeated three times, and subsequently, samples were recovered by spinning at 107 rcf for 2 min and re-diluted with TRIS buffer to the initial volume.

### 4.4. Acceptor Liposome Preparation

Liposomes were prepared by dispersing a thin lipid film made of egg phospholipids with 80% phosphatidylcholine (Lipoid E80, Lipoid GmbH, Ludwigshafen, Germany) according to a protocol for preparing 300 nm liposomes described by Hinna et al. [10]. The hydrated thin lipid film was extruded 21 times through 400 nm pore size; the large liposomes were then subjected to ten freeze–thaw cycles in dry ice/ethanol and 50 °C water bath, followed by 21 additional filter extrusions. The dispersion was diluted to a concentration of 10 mg/mL lipid, and an appropriate size fraction was collected by centrifugation for 120 min at 15,500× *g*. The obtained pellet was re-dispersed in the buffer while it was gently agitated on an orbital shaker and the centrifugation procedure was repeated to remove any remaining smaller vesicles.

### 4.5. Quantification of ZnPc, Phosphatidylcholine and Gd Chelate Content in Liposome Formulations

The amount of zinc phthalocyanine incorporated within liposomes was determined by UV-vis measurements using microplate reader FLUOstar Omega Microplate Reader (BMG LAB-TECH, Ortenber, Germany). All measurements were carried out in triplicate with measurements of the blank (DMF). A calibration curve was established from three independent measurement series at 670 nm. Non-loaded liposomes were measured under the same conditions to determine any potential interference of the lipids. POPC concentration in liposomal formulations was determined using the enzymatic assay Phospholipids (mti-diagnostics GmbH, Idstein, Germany). The test is based on a three-step enzymatic reaction employing phospholipase D, choline oxidase, and peroxidase [63]. Phospholipid standards, tested samples, and blank (2 µL of each) were transferred into 300 µL of chromogen solution and mixed in a 96-well plate. Subsequently, the plate was incubated at 37 °C for 15 min, and absorption was read against blank at 500 nm in a FLUOstar Omega Microplate Reader (BMG LAB- TECH, Ortenberg, Germany). There was no interference between ZnPc absorption and measurement wavelength of Phospholipids assay at 500 nm.

### 4.6. Dynamic Light Scattering (DLS) Measurements

DLS measurements were carried out at 20 °C. Per sample, 10 measurements were done with a data acquisition time of 10 s each. Measurements were done in backscattering mode (DelsaMax, Denmark ApS c/o, Copenhagen, Denmark) and analyzed with the DelsaMax software’s (Beckman Coulter, v. 1.0.1.6, Denmark ApS c/o, Copenhagen, Denmark). Results are given as z-average and polydispersity index (PDI) calculated by the instrument’s cumulant analysis.

### 4.7. Asymmetrical Flow Field-Flow Fractionation System with MALLS Detection

An Eclipse separation system (Eclipse 3+ AF4, Wyatt Technology Europe GmbH, Dernbach, Germany) was connected to a degasser, isocratic pump, and an autosampler with temperature control (Agilent 1200 series; Agilent Technology, Böblingen, Germany). A frit-inlet channel (Wyatt Technologies, Dernbach, Germany) was used, and a polyethersulfone membrane with a cut-off of 10 kDa served as the accumulation wall (Superon GmbH, Dernbach, Germany). The separation system was connected to MALLS (DAWN HELEOS II, Wyatt Europe, laser wavelength 658 nm). TRIS buffer (10 mM, pH 7.4), preserved (0.02% sodium azide) and filtered (pore size, 0.1 μm), was used as the carrier liquid.

### 4.8. Size Analysis by AF4 with MALLS Detection

Donor liposomes (10–20 µL) and acceptor liposomes (5 µL) were injected and fractionated under the conditions described in Table 3 (Method 1 and 2). Particle size and size distributions were calculated by the Astra software version 4.9 (Wyatt Europe, Dernbach, Germany) using the hollow sphere model. The data obtained from the detector at the lowest angle were excluded in all calculations because of the generally high noise. The obtained size results are given as mean geometric diameters determined over the peak area, where Dz is the intensity-weighted mean diameter. Size distributions are described using the percentile diameters (D10 and D90) and the median diameter (D50) obtained from the cumulative mass fractionation with an applied sigma spread factor of 10.

### 4.9. Transfer Study and Offline Quantification of ZnPc

To determine the ZnPc transfer kinetics from donor to acceptor liposomes, the DL and AL were mixed in a 1:0.8 mass ratio of DL-to-AL in a glass vial and stirred with a magnetic stirrer at 130 rpm, 37 °C. At different time points (up to 48 h), 200 µL of incubation mixtures were injected into the AF4 channel for fractionation under the conditions presented in Table 3 (Method 3 and 4). Subsequently, fractions of DL and AL were collected into 15 mL tubes. The collected fractions were up-concentrated in Vacuum Rotary Concentrator RVC-2-18 CD plus (Martin Christ GmbH, Osterode am Harz, Germany). The remaining residue was dissolved in DMF/MeOH mixture 3:1 *v/v* and centrifuged at 250 rpm, and the supernatant was transferred into a quartz microplate. Offline quantification of ZnPc in collected fractions DL and AL was performed by UV/Vis measurements in a FLUOstar Omega Microplate Reader (BMG LAB- TECH, Ortenberg, Germany) at 671 nm. ZnPc contents in collected DL and AL fractions were calculated from the calibration curve established for series of ZnPc dilutions in DMF/MeOH 3:1 *v/v*.

ZnPc released to the aqueous phase during fractionation was determined from the total donor and acceptor fraction recovery relative to the known initial content in the incubation mixture. The amounts of ZnPc transferred over time (loss from donor fraction and uptake into acceptor fraction) were fitted with the exponential function shown in Equation 1 using Sigma Plot 11 software:

(1) $$Y=Y_{eq}-Ae^{-kt};$$

where **Y** is the relative amount of ZnPc transferred between liposomal fractions at time **t** and **Y_eq_** is the relative amount transferred at equilibrium and marks the height of the plateau. **A** is the pre-exponential coefficient and **k** is the rate constant of transfer. Half-lives (**t_1/2_**) were determined from the obtained rate constants of transfer:

(2) $$t_{1/2}=\frac{ln2}{k}$$

The mechanism of ZnPc release from donor liposomes was analyzed using DDSolver software by fitting the obtained results with different kinetic models: Higuchi, Krosmeyer–Peppas, and Peppas–Sahlin [37].

## 5. Conclusions

A flow field-flow fractionation method was applied to study (i) the size and size distribution of theranostic nanovesicles and (ii) the transfer of a hydrophobic photosensitizer from theranostic nanoliposomes to acceptor liposomes. The latter served as an external sink, mimicking the in vivo situation when ZnPc is released from liposomal vesicles and immediately transferred to various biological sinks, mostly lipoproteins. The determined rate constant of ZnPc loss from theranostic liposomes allowed ranking among studied formulations prepared by TLH+extrusion (F4M1) and microfluidic method (F4M2 and F5M2). Theranostic vesicles prepared by microfluidics were 2–3 times smaller and had a higher D/L ratio than those obtained by extrusion. The release rates of ZnPc transfer from the formulations studied here were proportional to the liposome size and inversely proportional to the ZnPc/POPC molar ratio, meaning that ZnPc was retained longer in smaller liposomes and with a higher ZnPc/POPC ratio.

In essence, the AF4-based release/transfer analysis employed here can be considered a useful tool for comparing the release/transfer characteristics of different liposome formulations. The observed results appear to indicate that the method is suited for predictive formulation ranking.

## Figures and Tables

**Table 1:** Studied liposome samples, preparation methods, liposome components, ZnPc load, and POPC concentration determined from the enzymatic assay.

Sample Name	Components	Molar Ratio	ZnPc Load (µg/mL)	Phosphatidylcholine Concentration from Enzymatic Assay (mg/mL)
Method 1: Thin lipid film hydration + extrusion				
F4M1	ZnPc/PE-DTPAGd/POPG/POPC	0.05/0.75/2/8	8.03 ± 0.06	5.1 ± 0.3
Method 2: microfluidic FRR 3:1, TFR 12 mL min^−1^				
F4M2	ZnPc/PE-DTPAGd/POPG/POPC	0.05/0.75/2/8	19.86 ± 0.93	4.6 ± 0.4
F5M2	ZnPc/PE-DTPAGd/PEG-PE/POPG/POPC	0.05/0.75/0.2/2/8	11.00 ± 0.12	5.8 ± 0.3
Method 3: microfluidic FRR 5:1, TFR 12 mL min^−1^				
F4M3	ZnPc/PE-DTPAGd/POPG/POPC	0.05/0.75/2/8	22.02 ± 0.99	3.2 ± 0.2
Method 4: microfluidic FRR 3:1, TFR 8 mL min^−1^				
F4M4	ZnPc/PE-DTPAGd/POPG/POPC	0.05/0.75/2/8	12.29 ± 0.26	2.8 ± 0.2
Method: TLH + freeze–thawing				
Acceptor liposomes	egg phosphatidylcholine (PC)	-	-	9.8 ± 0.2

**Table 2:** Size and size distribution of theranostic liposomes measured by AF4-MALLS and DLS. All sizes are reported in diameter.

Sample Name	AF4-MALLS		DLS					
	Peak 1 D_z_ (nm)	Peak 2 D_z_ (nm)	Regularization Analysis				Cumulants	
			Peak 1 (nm)	Intensity (%)	Peak 2 (nm)	Intensity (%)	Z_av_ (nm)	PDI
Method 1: TLH+extrusion								
F4M1	67.1 ± 5.1	123.2 ± 7.4	64.5	100.0%	no peak identified		58.8 ± 0.5	0.084 ± 0.020
Method 2: microfluidic FRR 3:1, TFR 12 mL min^−1^								
F4M2	25.3 ± 1.8	196.9 ± 5.6	20.2	80.9%	61.4	19.1%	24.2 ± 0.3	0.235 ± 0.002
F5M2	24.5 ± 2.4	185.9 ± 0.7	28.9	93.0%	297.3	7.0%	32.8 ± 0.4	0.164 ± 0.016
Method 3: microfluidic FRR 5:1, TFR 12 mL min^−1^								
F4M3	23.7 ± 0.4	no peak identified	20.3	91.6%	104.9	8.4%	22.1 ± 0.4	0.175 ± 0.018
Method 4: microfluidic FRR 3:1, TFR 8 mL min^−1^								
F4M4	31.0 ± 3.1	no peak identified	29.2	92.2%	153.7	7.8%	28.1 ± 0.3	0.163 ± 0.016
Method: TLH + freeze–thawing								
Acceptor liposomes	276.2 ± 0.8	no peak identified	49.4	0.6%	319.2	99.4%	302.9 ± 16.7	0.111 ± 0.023

**Table 3:** AF4-MALLS methods used for liposome sizing and transfer study with applied crossflow profile, injection volume, and laser power.

AF4-MALLS Method	Method 1: Donor Liposomes Size Measurement		Method 2: Acceptor Liposomes Size Measurement		Method 3: Separation of Donor and Acceptor Liposomes		Method 4: Separation of Donor and Acceptor Liposomes	
Focus flow (V_F_)	1 mL/min		1 mL/min		1 mL/min		1 mL/min	
Injection volume	10–20 µL		5 µL		200 µL		200 µL	
Crossflow parameters	Crossflow gradient (mL/min)	Duration	Crossflow gradient (mL/min)	Duration	Crossflow gradient (mL/min)	Duration	Crossflow gradient (mL/min)	Duration
Laser power	100%		100%		20%		20%	
Mode: Elution + Injection	1	for 2 min	1	for 2 min	1	for 1 min	2	for 1 min
	1–0.3	over 1 min	1–0.3	over 1 min	1–0.2	over 3 min	2–0.5	over 1 min
	0.3	for 17 min	0.3	for 3 min	0.2	for 12 min	0.5	for 10 min
	0.3–0.02	over 2 min	0.3–0.02	over 2 min	0.2–0.02	over 5 min	0.5–0.02	over 4 min
	0.02	for 5 min	0.02	for 8 min	0.02	for 5 min	0.02	for 5 min
	0.02–0	over 1 min	0.02–0	over 1 min	0.02–0	over 1 min	0.02–0	over 1 min
	0	for 5 min	0	for 5 min	0	for 13 min	0	for 9 min
Method duration	33 min		22 min		40 min		31 min	

**Table 4:** Key kinetic parameters of ZnPc transfer (loss) from theranostic DL.

	Sample	F4M1	F4M2	F5M2
Parameter				
Plateau Y_eq_ (%)		48.7 ± 2.6	46.6 ± 3.9	53.1 ± 2.0
Rate constant, k (min^−1^)		0.0058 ± 0.0015	0.0025 ± 0.0007	0.0043 ± 0.0008
Half-life, t_1/2_ (min)		119.5 ± 0.3	277.3 ± 0.3	161.2 ± 0.2
R^2^		0.9231	0.9366	0.9612

**Table 5:** Parameters obtained by fitting the cumulative release profile of ZnPc from DL formulations to three mathematical models (the highest values of R^2^ and the lowest AIC are presented as bold numbers).

Model	Parameter	Formulation		
		F4M1	F4M2	F5M2
**Higuchi;** $\frac{M_{t}}{M_{\infty }}=k_{H}t^{1/2}$				
	k_H_	0.985	0.959	0.870
	R^2^	0.912	0.955	0.984
	AIC	54.323	48.645	37.902
	T_25_ (min)	644.759	679.713	826.294
	T_50_ (min)	2579.036	2718.85	3305.175
**Korsmeyer-Peppas;** $\frac{M_{t}}{M_{\infty }}=k_{KP}t^{n}$				
	k_KP_	2.658	1.627	0.741
	*n*	0.363	0.428	0.522
	R^2^	0.969	0.968	0.985
	AIC	45.859	47.298	39.351
	T_25_ (min)	477.134	596.683	847.264
	T_50_ (min)	3213.824	3019.090	2197.589
**Peppas-Sahlin;** $\frac{M_{t}}{M_{\infty }}=k_{1}t^{m}+k_{2}t^{2m}$				
	k_1_	1.122	0.672	0.336
	k_2_	−0.007	−0.002	−0.001
	m	0.542	0.615	0.671
	R^2^	**0.985**	**0.983**	**0.991**
	AIC	**40.813**	**42.920**	**36.382**
	T_25_ (min)	429.097	532.850	779.341
	T_50_ (min)	None Calc	None Calc	None Calc

$M_{\infty }$ is the amount of drug at the equilibrium state (sometimes very close to the amount of drug contained in the dosage form at the beginning of the release process); $M_{t}$ is the amount of drug released over time **t**; $k_{H}$, $k_{1,\;}k_{2}$ are the release constant of Higuchi, Korsmeyer–Peppas, and Peppas–Sahlin and are constants of incorporation of structural modifications and geometrical characteristics of the system; n, m is the exponent of release, related to the drug release mechanism in function of time t; **R** is the correlation coefficient; **AIC** is the Akaike information criterion; **T_50_** is the time at which 50% of a drug is released [38].

## Supplementary Materials

The following are available online at https://www.mdpi.com/article/10.3390/ijms221910456/s1.
