# Supplementary material for: Application of Asymmetrical Flow Field-Flow Fractionation for Characterizing the Size and Drug Release Kinetics of Theranostic Lipid Nanovesicles
Source: Int J Mol Sci. 2021 Sep 28;22(19):10456. doi: 10.3390/ijms221910456 (PMC8508677; doi:10.3390/ijms221910456)
Supplement: Supplementary file 1 [file ijms-22-10456-s001.zip › ijms-1363045-supplementary.pdf]

SUPPLEMENTARY MATERIAL

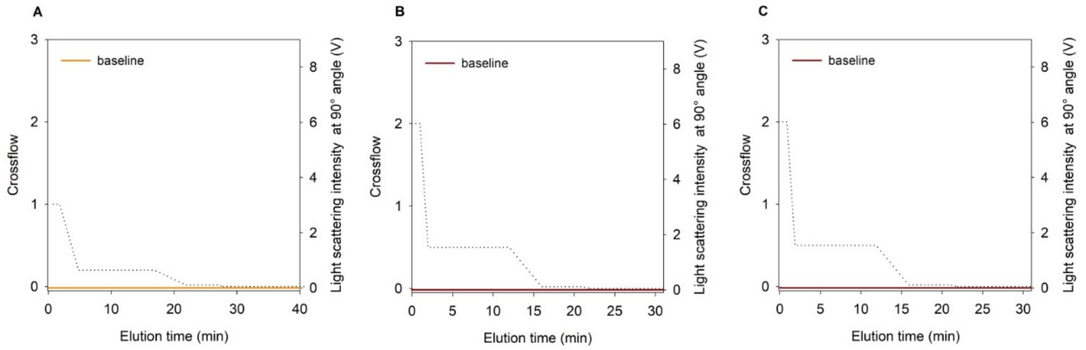

Figure S1 Baselines registered for blank injection of TRIS buffer under flow conditions (crossflow – dotted black line) used to separate donor and acceptor fractions: A - F4M1, B - F4M2, C – F5M2.

Table S1 Detailed results of size measurements of studied donor liposomes and acceptor vesicles by AF4/MALLS and DLS

Sample name	AF4/MALLS								DLS							
	Peak 1				Peak 2				Regularization analysis						Cumulants	
									Peak 1			Peak 2			Z_{av} (nm)	PDI
	D_{10} [nm]	D_{50} [nm]	D_{90} [nm]	D_z [nm]	D_{10} [nm]	D_{50} [nm]	D_{90} [nm]	D_z [nm]	Peak (nm)	Intensity (%)	PD(%)	Peak (nm)	Intensity (%)	PD(%)		
Method 1: TLH + extrusion																
F4M1	56.8±4.3	62.6±3.9	72.5±5.1	67.1±5.1	98.5±15.1	124.5±12.9	148.3±13.7	123.2±7.4	64.5	100.0	25.2	no peak identified			58.8±0.5	0.084±0.020
Method 2: microfluidic FRR 3:1, TFR 12 mL min^{-1}																
F4M2	27.1±4.8	45.3±6.7	58.9±3.5	12.7±1.8	153.9±1.5	189.3±0.6	212.5±6.4	196.9±5.6	20.2	80.9	6.2	61.4	13.1	11.5	24.2±0.3	0.235±0.002
F5M2	18.2±1.1	29.2±8.7	30.8±7.6	24.5±2.4	135.3±5.8	182.9±0.7	201.7±1.8	185.9±0.7	28.9	93.0	22.9	297.3	7.0	16.9	32.8±0.4	0.164±0.016
Method 3: microfluidic FRR 5:1, TFR 12 mL min^{-1}																
F4M3	30.3±1.6	36.9±1.0	49.5±11.7	23.7±0.4	no peak identified				20.3	91.6	5.6	104.9	8.4	8.1	22.1±0.4	0.175±0.018
Method 4: microfluidic FRR 3:1, TFR 8 mL min^{-1}																
F4M4	21.2±0.4	36.9±0.4	52.2±0.9	31.0±3.1	no peak identified				29.2	92.2	29.7	153.7	7.8	22.1	28.1±0.3	0.163±0.016
Method: TLH+ freeze-thawing																
Acceptor (hollow-sphere model)	193.5±8.9	210.9±5.0	223.7±3.3	219.1±3.9	no peak identified				49.4	0.6	4.4	319.2	98.3	16.3	302.9±16.7	0.111±0.023
Acceptor (solid-sphere model)	237.2±3.2	257.0±1.3	276.8±2.5	276.2±0.8	no peak identified											
